# Association between periodontal disease and Alzheimer's disease: a scoping review

**DOI:** 10.3389/fnagi.2025.1588008

**Published:** 2025-10-15

**Authors:** Xiaocui Zhang, Xin Huang, Mengdie Chang

**Affiliations:** Department of Stomatology, Sichuan Clinical Research Center for Cancer, Sichuan Cancer Hospital & Institute, Sichuan Cancer Center, University of Electronic Science and Technology of China, Chengdu, China

**Keywords:** periodontal disease, Alzheimer's disease, scoping review, mild cognitive impairment, periodontitis

## Abstract

**Background:**

Alzheimer's disease (AD) is the most common type of dementia, with mild cognitive impairment (MCI) as its early reversible stage. Periodontal disease (PD) is a chronic inflammatory condition associated with systemic diseases. Recent studies suggest a potential link between PD and AD/MCI. This scoping review evaluates the existing evidence on the association between PD and AD and explores possible mechanisms.

**Methods:**

A literature search was conducted in PubMed, Embase, and Cochrane databases (September 2025), covering studies from 2004 to 2025. Human clinical studies, animal models, and *in vitro* experiments were included, while reviews were excluded. Two independent researchers performed study selection, data extraction, and quality assessment.

**Results:**

A total of 52 studies were included after screening 1,369 records. Among them, 25 clinical studies examined the PD-AD association, including case-control, cohort, and cross-sectional studies. Additionally, 24 studies investigated underlying mechanisms, and 3 animal studies assessed PD-related interventions for AD. Evidence suggests PD increases the risk of AD and accelerates cognitive decline. Potential mechanisms include amyloid-β (Aβ) and tau protein aggregation, neuroinflammation triggered by *Porphyromonas gingivalis* (*Pg*) infection, and gut-brain axis dysregulation. Periodontal treatment and probiotics may have protective effects against AD-related pathology.

**Conclusions:**

PD may be a modifiable risk factor for AD, and periodontal interventions could contribute to AD prevention and management. Further clinical studies are needed to confirm the therapeutic potential of targeting oral microbiota in AD.

## 1 Background

Alzheimer's disease (AD) is a progressive neurodegenerative disorder characterized by memory impairment, cognitive decline, and behavioral disturbances, representing the leading cause of dementia worldwide and imposing a substantial burden on patients, caregivers, and healthcare systems (Alzheimer's Association, 2016, 2019; Hu et al., 2021). Neuropathologically, AD is defined by the accumulation of extracellular β-amyloid (Aβ) plaques, the formation of neurofibrillary tangles composed of hyperphosphorylated Tau protein, and subsequent synaptic dysfunction and neuronal loss (Pritchard et al., 2017; Jungbauer et al., 2022; Li et al., 2022; Mao et al., 2022; Weber et al., 2023; Wereszczyński et al., 2023; Zhao et al., 2023). In addition, chronic neuroinflammation—mediated by activated microglia, astrocytes, and proinflammatory cytokines such as IL-1β, IL-6, and TNF-α–has been recognized as a central driver of disease progression (Mao et al., 2022; Weber et al., 2023; Syrjälä et al., 2012; Pazos et al., 2018; Li et al., 2023). Emerging evidence further suggests that systemic inflammatory conditions can exacerbate neurodegenerative processes, underscoring the importance of identifying peripheral sources of inflammation as potential risk factors for AD (Kamer et al., 2008).

Periodontal disease (PD) is a chronic, multifactorial inflammatory disorder initiated by dysbiosis of the oral microbiome (Nakajima et al., 2016). It is characterized by the formation of microbial biofilms that stimulate host immune responses, resulting in periodontal tissue destruction, alveolar bone resorption, and eventual tooth loss (Lu et al., 2018). At the cellular level, PD involves infiltration of neutrophils, macrophages, and lymphocytes into periodontal tissues, accompanied by the production of proinflammatory cytokines (IL-1β, TNF-α, IL-6), prostaglandin E2, reactive oxygen species, and matrix metalloproteinases, which perpetuate tissue damage (Mirnic et al., 2024). As the disease advances, dysregulated host immunity drives chronic systemic inflammation (Wang, 2015). Importantly, periodontal pathogens such as *Pg*, Tannerella forsythia, and Treponema denticola can persist intracellularly, evade host defenses, and disseminate via the bloodstream (Giannini et al., 2023). Through bacteremia and infected leukocytes, microbial products (e.g., lipopolysaccharides, gingipains, bacterial DNA) may translocate to distant organs, including the brain (Banks, 2016).

The concept of a “PD–brain axis” has therefore attracted increasing attention as a plausible mechanistic link between oral and neurological health (Zhang et al., 2025). Chronic periodontal inflammation elevates systemic inflammatory burden (Murakami et al., 2013), disruption of the blood–brain barrier (Furutama et al., 2020), and priming of microglial responses (Mao et al., 2022). Circulating cytokines derived from periodontal lesions may enter the central nervous system (CNS) and amplify neuroinflammatory cascades (Liu et al., 2023). Furthermore, periodontal pathogens and their virulence factors have been detected in the brain tissue of patients with AD and other neurodegenerative conditions (Olsen, 2021). In particular, *P*g and its proteolytic enzymes, gingipains, have been shown to degrade neuronal proteins, promote Aβ aggregation, and enhance Tau hyperphosphorylation, thereby contributing to hallmark AD pathology (Jungbauer et al., 2022; Mao et al., 2022).

Collectively, these observations suggest that PD, through both systemic inflammation and microbial dissemination, may influence the onset and progression of AD. Considering that AD is a continuum with early preclinical changes occurring decades before clinical symptoms. Mild cognitive impairment (MCI), a condition in which cognitive function is lower than expected due to physiological aging, is a concept proposed by Petersen et al. in 1995. It is an intermediate state between normal cognition and predementia. It has also been identified as the first clinical stage of AD (Jia et al., 2020). The ability to carry out daily activities in the MCI state is still normal. A systematic review revealed that 32% of MCI patients will progress to AD within 5 years (Ward et al., 2013). However, this process is reversible (Rao et al., 2023). Interventions that eliminate related risk factors or enhance preventive factors in patients with MCI can effectively prevent AD (Nakamura et al., 2021). Understanding how periodontal inflammation interacts with neurodegenerative mechanisms is of critical importance. This scoping review aims to provide a comprehensive overview of current evidence linking PD to AD, highlight biological mechanisms underlying their interconnection, and discuss the implications of periodontal health management in the context of dementia prevention and therapy.

## 2 Methods

This study was designed as an exploratory scoping review with the primary aim of mapping and synthesizing the existing body of research on the association between PD and AD. The intention was not to assess intervention effectiveness or conduct meta-analyses but rather to provide a comprehensive overview of the types of evidence, study designs, and proposed mechanisms in this research field. Given its non-interventional nature and the absence of plans for a meta-analysis, formal registration (e.g., in PROSPERO) is not considered essential for this type of review. Nevertheless, to ensure transparency, the review protocol and the PRISMA-ScR Checklist have been included as Supplementary material (Appendix 1 and Appendix 2, respectively).

### 2.1 Data sources

A comprehensive literature search was conducted in September 2025. The databases searched included PubMed, EMBASE, and the Cochrane Central Register of Controlled Trials to minimize publication bias.

### 2.2 Search strategy

The following search strategy in PubMed utilized both keyword terms in the title and abstract fields as well as in the Medical Subject Headings (MeSH) to identify possible qualifying articles: (Periodontal Diseases[Mesh] OR Disease, Periodontal[Title/Abstract] OR Diseases, Periodontal[Title/Abstract] OR Periodontal Disease[Title/Abstract] OR Parodontosis[Title/Abstract] OR Parodontoses[Title/Abstract] OR Pyorrhea Alveolaris[Title/Abstract] OR Periodontitis"[Mesh] OR Periodontitides[Title/Abstract]) OR Pericementitis[Title/Abstract]) OR Pericementitides[Title/Abstract]) AND (Alzheimer Disease[Mesh] OR Alzheimer Dementia[Title/Abstract] OR Alzheimer Dementias[Title/Abstract] OR Dementia, Alzheimer[Title/Abstract] OR Alzheimer's disease[Title/Abstract] OR Dementia, Senile[Title/Abstract] OR Senile Dementia[Title/Abstract] OR Dementia, Alzheimer Type[Title/Abstract] OR Alzheimer Type Dementia[Title/Abstract] OR Alzheimer-Type Dementia (ATD)[Title/Abstract]). This search was translated and updated for Embase and the Cochrane Central Register of Controlled Trials accordingly (Sabharwal et al., 2021). When articles were on the topic of associations between PD and AD, the reference lists of included studies were manually searched, and citations of all included studies were checked to ensure search completeness (Dickson-Swift et al., 2022).

Studies were deemed eligible if they met the following criteria: (i) original clinical or experimental investigations conducted in human participants or animal models; (ii) explicitly examined the association between periodontal disease and Alzheimer's disease or explored potential underlying mechanisms; and (iii) published in English between January 2004 and February 2024. Excluded from consideration were systematic reviews, narrative reviews, and conference abstracts without full-text availability.

### 2.3 Data flustering

The search results were then saved and exported into EndNote, a bibliographic software program, to store, organize, and manage all the results (Dickson-Swift et al., 2022). After removal of duplicates, titles were examined by one author, and articles unrelated to PD and AD were removed. For the retained articles, clinical human and animal studies in which associations between PD and AD were explored or a potential mechanism was elucidated were included. Systematic and retrospective reviews were excluded.

Individual studies were tabulated, and brief descriptions of the following parameters were provided: name of the first author, year of publication, number of participants, country of study participants, study design, study population (human or animal), objective of the study, and outcomes, including statistical parameters and conclusions (Tables 1–3).

**Table 1:** Summary of epidemiological studies investigating the association between periodontal disease and Alzheimer's disease.

**Study**	**Objectives and study design**	**Study type**	**Number of participants**	**Location of study**	**Outcomes and conclusions**
Ma et al. (2022)	Identify the relationship between the longitudinal risk of developing Periodontal disease (PD) in a cohort of patients with dementia and Alzheimer's disease (AD) who did not show any signs of PD at baseline.	Retrospective cohort study	1,212	China	Outcomes: The incidence of PD in the AD group was significantly higher than that in the non-AD group [RR = 1.531, adjusted hazard ratio (aHR) = 1.667)]. Conclusions: AD were associated with a higher risk of PD dependent of age and independent of systemic confounding factors.
Schwahn et al. (2022)	Investigate the relationship between periodontal treatment and preclinical AD.	Quasiexperimental	586	Pomerania	Outcomes: Periodontal treatment had a favorable effect on AD-related brain atrophy (OR = −0.41). Conclusions: Periodontitis is related to preclinical AD.
Choi et al. (2019)	Investigate the association of Chronic Periodontitis on AD.	Retrospective cohort study	262,349	Korean	Outcomes: Compared with non-chronic periodontitis participants, chronic periodontitis patients had elevated risk for AD (aHR = 1.05). Conclusions: Chronic periodontitis may be associated with a higher risk of developing AD.
Carballo et al. (2023)	Assess whether periodontitis is associated with cognitive decline and its progression with certain blood-based markers of AD.	Prospective cohort study	101	Spain	Outcomes: Periodontitis was associated with poor cognitive performance and progression of cognitive impairment (hazard ratio [HR] = 1.8). The baseline levels of *p-*Tau (*p* < 0.001) and amyloid-β (Aβ) 1–40 (*p =* 0.036) in periodontitis patients were significantly higher than those in non-periodontitis patients. The concentration of Aβ protein increased with time in the periodontitis group (*p =* 0.005). Conclusions: Periodontitis is associated with cognitive decline. In addition, overexpression of *p-*Tau and Aβ may play a role in this association.
Fu et al. (2023)	Investigate the association between AD and periodontitis in the aspects of periodontal status, serological markers, and oral microbiome.	Case–control study	40	China	Outcomes: AD patients with Clinical Dementia Rating (CDR) ≥1 exhibited significantly more clinical attachment loss (CAL) than those with lower CDR. Conclusions: Periodontal infection is associated with AD.
Ide et al. (2016)	To investigate whether periodontitis is associated with increased severity of dementia, decreased cognitive function and increased systemic pro-inflammatory state in patients with AD.	Cohort study	60	United Kingdom	Outcomes: The presence of periodontitis was associated with a six fold increase in the rate of cognitive decline as assessed by the AD over a six month follow up period. Periodontitis at baseline was associated with a relative increase in the pro-inflammatory state over the six monthes follow up period. Conclusions: Periodontitis is associated with an increase in cognitive decline in AD, which may be mediated through effects on systemic inflammation.
Panzarella et al. (2022)	Evaluate the oral health status and its relationship with cognitive impairment of participants.	Case–control study	60	Italy	Outcomes: AD (*p =* 0.001) were positively correlated with the decayed, missing, and filled teeth. In addition, the presence of *Fusobacterium nucleatum* was significantly higher in AD than in controls (*p =* 0.02). Conclusions: AD is associated with chronic periodontitis, which is capable of determining tooth loss due to the pathogenicity of *Fusobacterium nucleatum*.
Noble et al. (2014)	Evaluate serum IgG to periodontal microbiota as possible predictors of incident AD.	Case-cohort study	219	USA	Outcomes: High anti-A. naeslundii titer was associated with increased risk of AD (HR = 2.0). High anti-E. nodatum IgG was associated with lower risk of AD (HR = 0.5). Conclusions: Serum IgG levels to common periodontal microbiota are associated with risk for developing incident AD.
Syrjälä et al. (2012)	Study the association between diagnosed dementia and oral health.	Cross-sectional study	354	Finland	Outcomes: Patients with AD had an increased likelihood of having teeth with deep periodontal pockets compared with non-demented persons. Conclusions: Patients with AD are at increased risk of periodontal diseases.
Yoo et al. (2023)	Investigate the associations of dental diseases and oral hygiene care with the risk of dementia.	Retrospective cohort study	2,555,618	Korean	Outcomes: Periodontal diseases was associated with an increased risk of all-cause dementia (aHR = 1.07). The increased risks by dental diseases was reduced by oral hygiene care (aHR = 0.94). Conclusions: Periodontal disease was independently associated with a higher risk of dementia. Conversely, improved oral hygiene care may modify the risk of dementia associated with dental diseases.
Laugisch et al. (2021)	Compare the periodontal and dental status in patients with either AD or other forms of dementia.	Cross-sectional study	20	Germany	Outcomes: Both patients with AD and other forms of dementia had periodontal disease. Conclusions: Patients with all forms of dementia (AD/other) need special dental care to improve periodontal and oral health.
Saito et al. (2022)	This study examined the relationship between the use of dental care among older people and the incidence of dementia based on health insurance claims data.	Cross-sectional study	31,775	Japan	Outcomes: Regarding the days of periodontal treatment, participants with ≥5 days had significantly lower aHRs for AD than those with 0 days (aHR = 0.88). Conclusions: Individuals who received periodontal treatment on many days had a low risk of AD.
Kamer et al. (2009)	Compare the differences of TNF-alpha and elevated antibodies to periodontal bacteria in AD and normal controls.	Case–control study	34	USA	Outcomes: Plasma TNF-alpha and antibodies against periodontal bacteria were elevated in AD patients compared with normal controls. and independently associated with AD. Conclusions: TNF-alpha and elevated numbers of antibodies against periodontal bacteria associate with AD and contribute to the AD diagnosis.
Na et al. (2024)	Evaluate the association between oral microbes and AD in periodontitis.	Case–control study	29	Korea	Outcomes: Differential analysis showed subgingival samples of the AD group had higher prevalence of Atopobium rimae, Dialister pneumosintes, *Olsenella* sp. HMT 807, *Saccharibacteria* (TM7) sp. HMT 348 and several species of Prevotella than the control group. Subgingival microbiome network analysis revealed a distinct, closely connected network in the AD group comprised of various Prevotella spp. and several anaerobic bacteria. Conclusions: A unique microbial composition was discovered in the subgingival region in the AD group. Potential periodontal pathogens were found to be more prevalent in the subgingival plaque samples of the AD group. These bacteria may possess a potential to worsen periodontitis and other systemic diseases.
Merchant et al. (2024)	Examine associations between empirically derived groups of 19 IgG antibodies against periodontal microorganisms and AD mortality.	Cross-sectional study	160	USA	Outcomes: With up to 21 years of follow-up, 160 AD-related deaths were documented. In the multivariable-adjusted model, AD mortality overall was not associated with IgG antibodies against periodontal microorganisms. Conclusions: Clusters of IgG antibodies against periodontal microorganisms did not predict AD mortality.
Karaduran et al. (2023)	Investigate the effect of periodontitis and current occlusal relationship on the progression rate of AD.	Prospective cohort study	90	Turkey	Outcomes: Stage II and Stage III toothed AD patients had higher percentage of bleeding on probing (BOP%) and clinical attachment level values than Stage I patients (*p* < 0.05). Stage III AD patients had significantly higher probing pocket depth (PPD) values than Stage I individuals (*p* < 0.05). Standardized Mini-Mental Test values showed positive correlation with BOP% (*r =* 0.308, *p =* 0.013) and PPD (*r =* 0.275, *p =* 0.027). Among the evaluated parameters, being in the AD Stage II-Stage III, having periodontitis and age variable had significant effects on Standardized Mini-Mental Testlevels (*p* < 0.05). Conclusions: Periodontitis may increase the severity and accelerate the progression rate of AD.
Sparks Stein et al. (2012)	Compare serum antibody levels to bacteria of periodontal disease in AD and control subjects. Compare serum antibody levels to bacteria of periodontal disease in AD and control subjects.	Case–control study	158	USA	Outcomes: Antibody levels to F nucleatum and P intermedia were significantly increased (*α =* 0.05) at baseline serum draw in the patients with AD compared with controls. Conclusions: In the years before the cognitive impairment, subjects had elevated antibodies to periodontal bacteria. Periodontal disease could contribute to the risk of AD onset/progression.
Sun et al. (2020)	Apply a two-sample Mendelian randomization (MR) approach to examine the potential causal relationship between chronic periodontitis and AD.	Bidirectional Mendelian randomization study	117,386	/	Outcomes: There was no association of genetically predicted AD with the risk of periodontitis (OR 1.00). Conclusions: Did not find convincing evidence to support periodontitis being a causal factor for the development of AD.
Martande et al. (2014)	Compare periodontal health status in individuals with and without AD.	Case–control study	118	India	Outcomes: All the evaluated periodontal parameters were higher in individuals with AD than that in cognitively normal individuals, and the periodontal status deteriorated with the progression of AD. There were significant differences in mean Gingival index, plaque index, probing depth, clinical attachment level, and percentage of bleeding sites between all the groups. Conclusions: The periodontal health status of individuals with AD deteriorates with disease progression and was closely related to their cognitive function.
Chen et al. (2017)	To determine whether patients with PD are at increased risk of developing AD	Matched-cohort study	27,963	China	Outcomes: Patients with 10 years of PD exposure exhibited a higher risk of developing AD than unexposed groups (aHR = 1.707). Conclusions: 10-year PD exposure was associated with a 1.707-fold increase in the risk of developing AD.
Holmer et al. (2018)	To test whether PD contributes to increased risk of mild cognitive impairment (MCI), subjective cognitive decline and AD.	Case–control study	154	Sweden	Outcomes: Poor oral health and marginal alveolar bone loss were more prevalent among MCI and AD patients than normal controls. The MCI and AD group was associated with generalized marginal alveolar bone loss (O*R =* 5.81), increased number of deep periodontal pockets (OR = 8.43;) and dental caries (OR = 3.36). Conclusions: Marginal periodontitis is associated with early cognitive impairment and AD.
Beydoun et al. (2020)	Examine associations of clinical periodontal and bacterial parameters with incident all-cause and AD dementia as well as AD mortality.	Cross-sectional study	33,199	USA	Outcomes: Among those ≥65 years, AD incidence and mortality were consistently associated with probing pocket depth, two factors and one cluster comprised of IgG titers against *Pg, Prevotella melaninogenica* (*P. melaninogenica*) and Campylobacter rectus (C. rectus) among others. Specifically, AD incidence was linked to a composite of C. rectus and *Pg* titers (aHR = 1.22), while AD mortality risk was increased with another composite (aHR = 1.46) loading highly on IgG for *Pg, Prevotella intermedia, Prevotella nigrescens, Fusobacterium nucleatum*, C. rectus, *Streptococcus intermedius, Capnocylophaga Ochracea*, and *P. melaninogenica*. Conclusions: Periodontal pathogens are associated with AD, which was stronger for older adults.
Rubinstein et al. (2024)	To examine the association of clinical, microbiological, and host response features of periodontitis with MRI markers of atrophy/cerebrovascular disease. Clinical periodontal data, microbial plaque, serum samples, and brain MRIs were analyzed.	Cross-sectional study	468	USA	Outcomes: More teeth were associated with lower odds of infarcts, lower white matter hyperintensity volume, higher entorhinal cortex volume, and higher cortical thickness. Higher extent of periodontitis was associated with lower entorhinal cortex volume and cortical thickness. Specific bacterial colonization and serum IgG responses showed differential associations with MRI outcomes. Conclusions: Clinical, microbiological, and serological features of periodontitis are associated with MRI findings related to Alzheimer's disease-related dementia risk; causal links need further study.
Hu et al. (2024)	To investigate the causal relationship between periodontal disease and AD using two-sample Mendelian randomization (MR). Periodontal disease data were obtained from the FinnGen database, and two sets of AD data were obtained from the IEU consortium and PGC databases.	Mendelian randomization study	1,814,363	/	Outcomes: Random-effects IVW analysis showed no evidence of genetic causal relationship between periodontal disease and AD using either IEU or PGC AD data. No heterogeneity, pleiotropy, or outliers were observed. Conclusions: There is no causal relationship between periodontal disease and AD at the genetic level.
Mo et al. (2025)	To explore the causal relationship between gingivitis, periodontitis, and AD using MR combined with bioinformatics, and to identify potential diagnostic biomarkers. Exposures/outcomes were derived from GWAS datasets. Transcriptome datasets from GEO were analyzed to identify key pathways, functions, and gene signatures.	Mendelian randomization study	199,807	/	Outcomes: inverse variance-weighted analysis showed positive association between chronic gingivitis and early-onset AD (O*R =* 1.161), validated by MR-Egger (O*R =* 1.296). Reverse analysis was negative. Immune activation, angiogenesis, and blood-brain barrier damage identified as shared mechanisms. Inter-alpha-trypsin inhibitor heavy chain H5 (ITIH5) gene and TGFB pathway highlighted; diagnostic gene signature (ITIH5, MFAP4, PRELP) shows potential for early-onset AD diagnosis. Conclusions: Chronic gingivitis may increase early-onset AD risk. Gene signature and pathways may serve as biomarkers and therapeutic targets.

**Table 2:** Mechanistic insights into the relationship between periodontal disease and Alzheimer's disease: evidence from human and experimental studies.

**Study**	**Objectives and study design**	**Study type**	**Number of participants**	**Location of study**	**Outcomes and conclusions**
Leblhuber et al. (2020)	Investigate the correlation between oral pathogens and AD.	Cross-sectional study	20	Austria	Outcomes: The presence of *Pg*, the key pathogen and one of the species involved in chronic periodontitis, was found to be associated with lower mini mental state examination scores (*p* < 0.05) and with a tendency to lower scores in the clock drawing test (*p =* 0.056). In addition, association between lower serum concentrations of the immune biomarker neopterin and the presence of Treponema denticola (*p* < 0.01) as well as of kynurenine were found in AD patients positive vs. negative for *Tannerella forsytia* (*p* < 0.05). Conclusions: Periodontal pathogens may be associated with cognitive impairment, *Treponema denticola* and *Tannerella forsytia* may alter the host immune response in AD. An altered salivary microbiome may be a causal link between chronic periodontitis and cognitive impairment in AD.
Poole et al. (2013)	Establish a link between PD and AD.	Case–control study	20	UK	Outcomes: Lipopolysaccharide (LPS) from *Pg* were positive when screened by immunofluorescence in 4 AD brain specimens. However, all controls remained negative (*p =* 0.029). Conclusions: LPS from periodontal bacteria can access the AD brain during life.
Qiu et al. (2024)	Aim to characterize both the microbial community of subgingival plaque and the metabolomic profiles of gingival crevicular fluid in patients with AD and mild cognitive impairment (MCI).	Cross-sectional study	96	China	Outcomes: The severity of periodontitis was significantly increased in AD patients compared with MCI patients and cognitively normal people. 19 differentially abundant metabolites were significantly correlated with *Veillonella parvula, Dialister pneumosintes, Leptotrichia buccalis, Pseudoleptotrichia goodfellowii*, and *Actinomyces massiliensis*, in which galactinol, sn-glycerol 3-phosphoethanolamine, D-mannitol, 1 h-indole-1-pentanoicacid, 3-(1-naphthalenylcarboy)- and L-iditol yielded satisfactory accuracy for the predictive diagnosis of AD progression. Conclusions: Periodontal microbial dysbiosis and metabolic disorders may be involved in the etiology and progression of AD.
Kubota et al. (2014)	Compare differences of AD pathway molecules in healthy tissues and periodontitis tissues, including amyloid beta (A4) precursor protein (APP), a key gene in AD, interleukin-1 beta (IL-1β), and complement component 1 (q subcomponent, A chain) (C1QA).	Case–control study	28	Japan	Outcomes: APP, IL-1β, and C1QA mRNA levels were significantly upregulated in periodontitis-affected gingival tissues. Conclusions: Elevated APP, IL-1β, and C1QA transcripts and APP-expressing macrophages in periodontitis-affected gingival tissues were observed, suggesting a relationship between periodontitis and AD pathogenesis.
Kamer et al. (2015)	Investigate whether peripheral inflammatory and/or infectious conditions in humans can promote Aβ brain accumulation.	Cross-sectional study	38	USA	Outcomes: Clinical attachment loss (≥3 mm), representing a history of periodontal inflammatory/infectious burden, was associated with increased C-Pittsburgh compound B uptake in Aβ vulnerable brain regions (*p =* 0.002). Conclusions: There is a correlation between periodontal disease and brain Aβ load.
Gil-Montoya et al. (2025)	Investigate association between periodontitis and cerebral Aβ accumulation in MCI patients.	Observational study	164	Spain	Outcomes: Patients with moderate-to-severe periodontitis exhibited a significantly higher risk of abnormal cerebral Aβ accumulation. The odds of Aβ positivity were 3.30 times higher for MCI/Aβ+ vs. MCI/Aβ- groups, and 4.94 times higher for MCI/Aβ+ vs. non-MCI/Aβ- participants. Conclusions: Periodontal disease may be associated with abnormal cerebral Aβ deposition in older adults, independent of cognitive status.
Sato et al. (2022)	Cells research describes the effect of *Pg* LPS on the expression of interleukin-6 (IL-6) and C-C motif chemokine ligand 2 (CCL2) in cultured hCMEC/D3 human brain microvascular endothelial cells.				Outcomes: *Pg* LPS-induced mRNA and protein expression of IL-6 and CCL2 in hCMEC/D3 cells in a concentration-dependent manner at the concentration of 0.5-50 μg/mL. Induction of IL-6 and CCL2 by *Pg* LPS was almost completely inhibited by pretreatment of cells with TLR4 inhibitor but not by TLR2 inhibitor. Treatment of cells with *Pg* LPS induced phosphorylation of nuclear factor-κB (NF-κB) p65, p38 mitogen-activated protein kinase (MAPK) and c-Jun N-terminal kinase (JNK). IL-6 induction was decreased by pretreatment of cells with NF-κB inhibitor SN50 or p38 MAPK inhibitor SB203580, while CCL2 induction was reduced by SN50 or JNK inhibitor SP600125. Conclusions: IL-6 and CCL2 produced upon *Pg* LPS stimulation may contribute to the inflammatory reactions in brain endothelial cells and subsequent neurological disorders such as cerebrovascular and AD.
Díaz-Zúñiga et al. (2019)	Cell research determine the effects of different serotypes of (a, b or c) aggregatibacter actinomycetemcomitans (Aa) LPS on primary cultures of microglia or mixed hippocampal cells.				Outcomes: Both culture types exhibited higher levels of inflammatory cytokines (IL-1β, IL-6 and TNFα) when treated with serotype b-LPS, compared with controls. Additionally, cultures treated with serotype a-LPS displayed increased mRNA levels of the modulatory cytokines IL-4 and IL-10. Mixed hippocampal cultures treated with serotype b-LPS exhibited severe neuronal morphological changes and displayed increased levels of secreted Aβ peptide. These results indicate that LPS from different Aa serotypes triggers discriminatory immune responses, which differentially affect primary hippocampal cells. Conclusions: Treatment with serotype b-LPS triggers the secretion of proinflammatory cytokines by microglia, induces neurite shrinking, and increases the extracellular Aβ evels, all features strongly associated with the etiology of AD.
[-2pt] Xue et al. (2020)	Animal study to investigate the causal relationship between PD and cognitive decline and the underlying mechanism in mice.				Outcomes: During the 12-month follow-up period of induce PD in mice. Severe alveolar bone loss and inflammatory changes were observed in gingival tissues, accompanied by progressive cognitive deficits. In addition, observed cerebral neuronal and synaptic injury and glial activation in this mouse model of PD. Furthermore, PD mice exhibited significant dysbiosis of the oral and gut microbiota, disruption of the intestinal barrier and blood–brain barrier, increases in the serum contents of proinflammatory cytokines and LPS, and increases in brain LPS levels, Toll-like receptor 4 (TLR4) expression, nuclear factor-κB (NF-κB) nuclear translocation and proinflammatory cytokine mRNA levels. Conclusions: PD may directly induce progressive cognitive decline and its mechanism is probably related to microbiota-gut-brain axis disorders, LPS/TLR4/NF-κB signaling activation and neuroinflammatory responses in mice. Therefore, the microbiota-gut-brain axis may provide the potential strategy for the prevention and treatment of PD-associated cognitive impairment.
Kantarci et al. (2020)	Animal study to test the impact of ligature-induced PD on 5xFAD mice and WT littermates.				Outcomes: PD increased the level of Iba1-immunostained microglia in WT mice. In 5xFAD mice, PD increased the level of insoluble Aβ. The increased level in Iba1 immunostaining that parallels the accumulation of Aβ in 5xFAD mice was not affected by PD except for a decrease in the dentate gyrus. A decline in Iba1 in the proximity of Aβ plaques in 5xFAD mice with PD compared to those without PD suggesting a PD-induced decrease in plaque-associated microglia (PAM). PD reduced IL-6, MCP-1, GM-CSF, and IFN-γ in brains of WT mice and reduced IL-10 in 5xFAD mice. Conclusions: PD increases neuroinflammation in WT mice and disrupts the neuroinflammatory response in 5xFAD mice and suggest that microglia is central to the association between PD and AD.
Gu et al. (2020)	Animal study to test whether periodontitis is involved in the exacerbation, contributing to AD pathologies.				Outcomes: Compared with control mice, bone loss in tibia (26% decrease) and memory decline (47% decrease) were induced in mice with a positive correlation after exposure to *Pg* (*p =* 0.0011). The IL-6 and IL-17 expression in tibia was negatively correlated with the bone volume/total tissue volume (*p =* 0.0052; *p =* 0.0019), while that in the cortex was negatively correlated with the memory test latency (*p =* 0.0017; *p =* 0.0351). Furthermore, the IL-17 expression in microglia was positively correlated with Aβ accumulation in neurons (*p* < 0.0001). In cultured MG6 microglia, the P gLPS-increased IL-6 expression was inhibited by a PI3K-specific inhibitor (68% decrease), and that of IL-17 was inhibited by IL-6 antibody (41% decrease). In cultured N2a neurons, conditioned medium from *Pg* LPS-stimulated microglia (MCM) but not *Pg* LPS increased the productions of APP, CatB, and Aβ, which were significantly inhibited by pretreatment with IL-17 antibody (67%, 51%, and 41% decrease). Conclusion: Chronic systemic exposure to *Pg* LPS simultaneously induces inflammation-dependent bone loss and AD-like pathologies by elevating IL-6 and IL-17 from middle age, suggesting that periodontal bacteria induce exacerbation of bone loss and memory decline, resulting in AD progression.
Ilievski et al. (2018)	Animal study to test whether repeated exposure of WT C57BL/6 mice to orally administered *Pg* results in neuroinflammation, neurodegeneration, microgliosis, astrogliosis and formation of intra- and extracellular amyloid plaque and neurofibrillary tangles (NFTs) which are pathognomonic signs of AD.				Outcomes: Significantly greater levels of expression of IL6, TNFα and IL1β were evident in experimental as compared to control group (*p* < 0.01, *p* < 0.00001, *p* < 0.00001, respectively). In addition, microgliosis and astrogliosis were evident in the experimental but not in control group (*p* < 0.01, *p* < 0.0001, respectively) amyloid APP and beta-site APP cleaving enzyme 1 gene expression were increased in experimental group compared with control group (*p* < 0.05, *p* < 0.001, respectively). A disintegrin and metalloproteinase domain-containing protein10 gene expression was significantly decreased in experimental group compared with control group (*p* < 0.01). Extracellular Aβ was detected in the parenchyma in the experimental but not in the control group (*p* < 0.00001). Finally, phospho-Tau (Ser396) protein was detected and NFTs were evident in experimental but not in the control group (*p* < 0.00001). Conclusions: Neurodegeneration and the formation of extracellular Aβ in young adult WT mice after repeated oral application of *Pg*, which suggest that low grade chronic periodontal pathogen infection can result in the development of neuropathology that is consistent with that of AD.
Qian et al. (2021)	Animal study to investigate the effect of periodontitis on learning capacity and memory of APP/presenilin (PS1) transgenic mice along with the mechanisms underlying these effects.				Outcomes: Mice in the *Pg* LPS Injection + Ligation group exhibited cognitive impairment and a significant reduction in the number of neurons. Glial cell activation in the experimental groups with significantly increased Aβ levels was more pronounced relative to the control group. Induction of periodontitis was concurrent with an increase in cyclooxygenase-2, inducible nitric oxide synthase, APP, and beta-secretase 1 expression and a decrease in A disintegrin and metalloproteinase domain-containing protein 10 expression. Conclusions: Periodontitis exacerbated learning and memory impairment in APP/PS1 mice and augmented Aβ and neuroinflammatory responses.
Lu et al. (2022)	Animal study to explore the influence of periodontitis-related salivary microbiota on AD based on the gut-brain crosstalk in APP/PS1 transgenic mice.				Outcomes: Continuous gavage of periodontitis-related salivary microbiota in PAP mice impaired cognitive function and increased β-amyloid accumulation and neuroinflammation. Moreover, these AD-related pathologies were consistent with gut microbial dysbiosis, intestinal proinflammatory responses, intestinal barrier impairment, and subsequent exacerbation of systemic inflammation, suggesting that the periodontitis-related salivary microbiota may aggravate AD pathogenesis through crosstalk of the gut-brain axis.
					Conclusions: Periodontitis might participate in the pathogenesis of AD by swallowing salivary microbiota, verifying the role of periodontitis in AD progression.
Yamada et al. (2020)	Cells research to evaluate the influence of phosphoglycerol dihydroceramide (PGDHC) on hallmark findings in AD.				Outcomes: *Pg*-derived PGDHC, but not *Pg*-LPS, upregulated secretion of soluble Aβ peptide and expression of APP in CHO-7WD10 cells. Furthermore, hyperphosphorylation of tau protein was observed in SH-SY-5Y cells in response to PGDHC lipid. In contrast, *Pg*-LPS had little, or no significant effect on the tau phosphorylation induced in SH-SY-5Y cells. However, both PGDHC and *Pg* LPS contributed to the senescence of SH-SY5Y cells as indicated by the production of senescence-associated secretory phenotype (SASP) markers, including beta-galactosidase, cathepsin B (CtsB), and pro-inflammatory cytokines Tumor Necrosis Factor α(TNF-α), and IL-6. Additionally, PGDHC diminished expression of the senescence-protection marker sirtuin-1 in SH-SY-5Y cells. Conclusions: *Pg*-derived PGDHC ceramide promotes amyloidogenesis and hyperphosphorylation, as well as the production of SASP factors. Thus, PGDHC may represent a novel class of bacterial-derived virulence factors for AD associated with periodontitis.
Bahar et al. (2021)	Animal study to investigate the effect of *Pg* (W83) oral infection on the development of AD pathophysiology in a wild-type obese, diabetic (db/db) mouse model.				Outcomes: Immunohistochemistry (glial cell markers) of the *Pg*-infected mice tissue sections exhibited neuroinflammation in the form of reactive microglia and astrocytes. Anti-tau immunopositivity, in addition to cells, was prominent in thickened axons of hippocampal CA neurons. The mRNA abundance of crucial genes in the insulin signaling pathway (INSR, IGF1, IRS, IDE, PIK3R, SGK1, GYS, GSK3B, AKT1) were upregulated, potentially exacerbating insulin resistance in the brain by *Pg* oral infection. Increased mRNA abundance of several kinases, membrane receptors, transcription factors, and pro-inflammatory mediators indicated hyperactivation of intracellular cascades with potential for tau phosphorylation and Aβ release in the same infection group. Conclusion: *Pg* W83 infection of db/db mice provides a disease comorbidity model with the potential to reproduce AD pathophysiology with induced periodontal disease.
Ma et al. (2023)	Animal study to investigate the effects of *Pg* and *Pg*-derived extracellular vesicles (pEVs) on periodontitis pathogenesis and cognitive impairment in mice. Cognitive function was assessed using Y-maze and novel object recognition tasks. Biomarkers were measured using ELISA, qPCR, immunofluorescence, and pyrosequencing.				Outcomes: pEVs contained neurotoxic gingipains, fimbria proteins, and LPS. Gingival exposure (not oral gavage) of *Pg* or pEVs caused periodontitis and memory-impairment–like behaviors. Increased TNF-α expression in gingival and hippocampal tissues, more GP+Iba1+, LPS+Iba1+, NF-κB+Iba1+ cells, decreased BDNF, claudin-5, NMDA receptor expression, and fewer BDNF+NeuN+ cells. F-pEVs detected in trigeminal ganglia and hippocampus; trigeminal neurectomy blocked transport. *Pg*/pEV exposure increased blood LPS and TNF-α, caused colitis and gut dysbiosis. Conclusions: Gingivally infected *Pg*, especially pEVs, may contribute to cognitive decline by translocating into the brain via the trigeminal nerve and bloodstream, inducing neuroinflammation and gut dysbiosis. pEVs may be an important risk factor for dementia.
Hao et al. (2022)	Animal study using an amyloid precursor protein knock-in AD mouse model to investigate whether oral infection with *Pg* aggravates AD pathology and cognitive impairment, and to explore the immune mechanisms involved.				Outcomes: Oral *Pg* infection worsened behavioral and cognitive deficits, and accelerated Aβ accumulation in AD mice. *Pg* infection enhanced neuroinflammation via Aβ-primed microglial activation and caused *Pg* entry into the brain. *Pg*-induced overactivation of complement C1q amplified microglial activation, promoted neuroinflammation, and tagged synapses for microglial engulfment. Conclusions: Periodontal infection significantly aggravates AD progression through innate immune pathways, suggesting that targeting microbial etiology and periodontal treatment may help ameliorate AD symptoms and reduce prevalence
Nie et al. (2019)	Animal and cell-based study using middle-aged mice and RAW264.7 macrophage cell line to investigate whether chronic systemic *Pg* infection contributes to Aβ accumulation in peripheral inflammatory tissues and subsequently in the brain.				Outcomes: Chronic systemic exposure to *Pg* LPS increased IL-1β, AβPP770, CatB, Aβ1-42, and Aβ3-42 in mouse liver, mainly co-localized with macrophages. *In vitro*, blocking CatB or NF-κB inhibited *Pg*-induced expression of IL-1β and Aβ-related proteins. Aβ3-42 induced significant macrophage death and stronger reduction of phagocytic ability than Aβ1-42. In gingival tissue macrophages from periodontitis patients, AβPP770, CatB, Aβ1-42, and Aβ3-42 were detected. Conclusions: Chronic systemic *Pg* infection promotes Aβ accumulation in monocytes/macrophages via CatB/NF-κB signaling, suggesting that monocytes/macrophages may act as a circulating Aβ pool in periodontitis patients. CatB may be a novel therapeutic target to prevent periodontitis-related AD initiation and progression.
Kong et al. (2025)	Animal study to investigate whether *Pg*-induced periodontitis triggers type I interferon responses and activates the IFITM3–Aβ axis, thereby contributing to AD–like pathology.				Outcomes: *Pg*-induced periodontitis led to cognitive impairment in C57BL/6J mice and exacerbated cognitive decline in APP/PS1 mice. It significantly increased levels of IFN-β, IFITM3, and Aβ deposition in the brain, and *Pg* DNA was detected alongside glial activation and inflammatory mediator expression. Astrocytes were confirmed as the primary responders to *Pg*-induced innate immunity both *in vitro* and *in vivo*. Periodontitis also induced IFITM3 expression in periodontal tissues and salivary glands. Conclusions: *Pg*-induced periodontitis aggravates cognitive impairment and promotes AD pathology by enhancing blood-brain barrier permeability, triggering neuroinflammation, and upregulating IFITM3 to promote Aβ deposition. These findings highlight the importance of periodontal disease prevention and treatment in reducing AD progression.
Yavuz et al. (2025)	Animal study to investigate the effects of experimentally induced periodontitis and non-surgical periodontal therapy on behavior, neurodegeneration, and neuroinflammation in rats with AD-like pathology.				Outcomes: Rats with AD and those with AD plus periodontitis showed significantly worse performance in behavioral tests compared with controls, whereas rats receiving non-surgical periodontal therapy demonstrated performance similar to controls. Cerebrospinal fluid phosphorylated tau levels were comparable between AD and AD plus periodontitis groups, but the hippocampal phosphorylated tau to total tau ratio was significantly higher in the AD plus periodontitis group. BACE1, NLRP3, and iNOS levels showed no significant differences among groups. Notably, non-surgical periodontal therapy resulted in a reduction of NF-κB levels. Conclusions: Periodontitis may exacerbate AD-like molecular pathology, particularly by promoting tau hyperphosphorylation, whereas non-surgical periodontal therapy appears to mitigate disease progression and improve behavioral outcomes.
Verma et al. (2025)	Cells research to investigate the role of caspase-4 in *Pg*-LPS-induced neuroinflammation, oxidative stress, and mitochondrial dysfunction.				Outcomes: Silencing caspase-4 significantly reduced IL-1β secretion by inhibiting the caspase-4–NLRP3–caspase-1–gasdermin D inflammasome pathway, confirming its key role in neuroinflammation. Caspase-4 knockdown decreased the activation of amyloid precursor protein and presenilin-1, reduced amyloid-β peptide secretion, and restored the expression of neuroinflammatory markers such as total tau, VEGF, TGF, and IL-6. Caspase-4 also modulated oxidative stress by reducing reactive oxygen species production and lowering oxidative stress markers (iNOS and 4-hydroxynonenal). Moreover, caspase-4 influenced mitochondrial membrane potential, biogenesis, fission, fusion, respiration, and ATP production — all of which were impaired by *Pg*-LPS but restored when caspase-4 was inhibited. Conclusions: Caspase-4 is a central mediator of *Pg*-LPS-induced neuroinflammation, amyloidogenesis, oxidative stress, and mitochondrial dysfunction, making it a potential therapeutic target for AD and related dementias.
Zhang et al. (2025)	Animal study to investigate the causal link between *Pg*, intestinal inflammation, and AD-like pathology through the microbiota–gut–brain axis.				Outcomes: Chronic oral exposure to *Pg* combined with ligature placement for 24 weeks impaired behavioral performance in open field, novel object recognition, and Y-maze tests. *Pg* infiltrated the brain, increasing Aβ42, AβPP, and Aβ fragments, promoting tau phosphorylation and microglial activation, while reducing ZO-1, PSD95, SYP, and NeuN levels. Inflammatory mediators (NLRP3, caspase-1, IL-1β, IL-6, TNF-α) were elevated in brain and intestine, whereas intestinal ZO-1 and occludin decreased. Gut microbiota composition was significantly altered. Conclusions: *Pg* induces gut dysbiosis, activates the NLRP3 inflammasome in brain and intestine, disrupts both intestinal and blood–brain barriers, triggers neuroinflammation, and promotes AD progression. This study highlights the pivotal role of the NLRP3 inflammasome in the microbiota–gut–brain axis in *Pg*-induced AD-like pathology.
Zhu et al. (2025)	Animal study to investigate the role of *Pg* infection and gut–brain axis in AD-like pathology.				Outcomes: Oral administration of Pginduced alveolar bone resorption, intestinal barrier impairment, and AD-like lesions. Infection caused oral and gut microbiota dysbiosis, disrupted the tryptophan metabolism pathway, and elevated 3-hydroxykynurenine levels in serum and hippocampus. The metabolite 3-hydroxykynurenine suppressed Bcl2 gene expression, leading to neuronal apoptosis and exacerbating AD-like pathology in both *in vivo* and *in vitro* models. Conclusions: *Pg* promotes AD pathogenesis through the microbiota–gut–brain axis via metabolic disturbance and neuronal apoptosis, providing new insights for AD prevention and treatment.

**Table 3:** Treatments for periodontal disease and their impact on Alzheimer's disease pathology.

**Study**	**Objectives and study design**	**Study type**	**Number of participants**	**Location of study**	**Outcomes and conclusions**
**Animal study on treatment of periodontal disease and Alzheimer's disease**					
Zhao et al. (2023)	Animal study to investigate the effect nisin (a class I *Lantibiotic bacteriocin* produced by the probiotic *Lactococcus lactis*) in modulating brain pathology triggered by periodontitis.				Outcomes: Nisin treatment mitigated the changes in the brain microbiome composition, diversity, and community structure, and reduced the levels of periodontal pathogen DNA in the brain induced by periodontal disease. Nisin treatment significantly decreased the mRNA expression of proinflammatory cytokines (IL-1β, IL-6, and TNF-α) in the brain that were elevated by periodontal infection. In addition, the concentrations of Aβ total Tau, and Tau were significantly higher in the infection group compared to the control group, respectively. Nisin treatment markedly reduced the Aβ, total Tau, and phosphorylated Tau deposition in the brain of the infection group. Conclusions: Nisin abrogation of brain microbiome dysbiosis induces beneficial effects on AD-like pathogenic changes and neuroinflammation, and thereby may serve as a potential therapeutic for periodontal–dysbiosis-related AD.
Dominy et al. (2019)	Animal study to investigate the effect of small-molecule inhibitors targeting gingipains (toxic proteases from *Pg*) on block *Pg* neurotoxicity.				Outcomes: Gingipain inhibition reduced the bacterial load of an established *Pg* brain infection, blocked production, reduced neuroinflammation, and rescued neurons in the hippocampus. Conclusions: Gingipain inhibitors could be valuable for treating *Pg* brain colonization and neurodegeneration in AD.
Liu et al. (2025)	Animal study to evaluate a targeted intracerebral antimicrobial nano-delivery system for *Pg*-induced Alzheimer's disease-like cognitive impairment.				Outcomes: A nano-delivery system was developed by coating macrophage membranes (stimulated by *Pg*) onto platinum nanoclusters (*Pg*-M-PtNCs). The spherical *Pg*-M-PtNCs (=50 nm) exhibited good biocompatibility, inhibited macrophage phagocytosis, and enhanced bacterial adherence. *In vitro, Pg*-M-PtNCs significantly inhibited *Pg* growth. *In vivo, Pg*-M-PtNCs were efficiently delivered to and retained at the brain infection site, reducing bacterial load and neuronal damage, thereby improving AD-like cognitive dysfunction in chronic periodontitis mice. Conclusions: Platinum nanoclusters coated with *Pg*-stimulated macrophage membranes effectively target intracerebral bacteria and improve cognitive impairment caused by *Pg* brain infection, highlighting a promising therapeutic strategy for AD associated with periodontal infection.

## 3 Results

**Figure 1:**
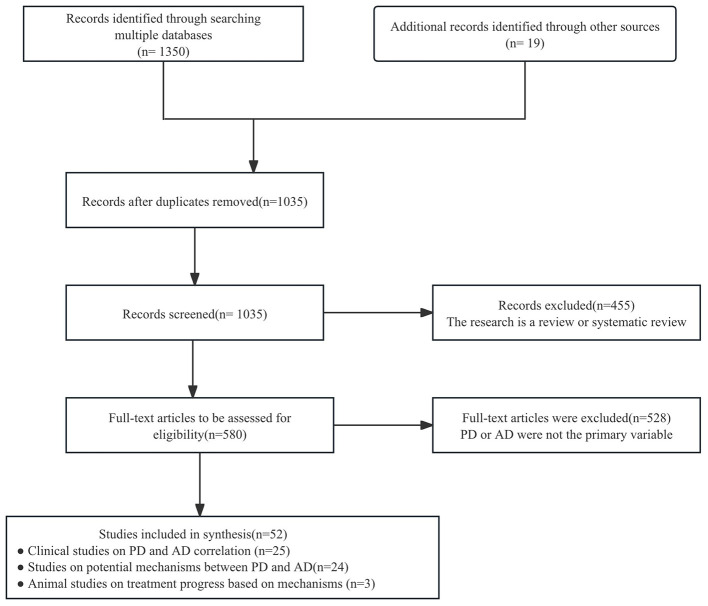
Flow of studies through the scoping review.

The initial search yielded 1,369 results. After deduplication, 1,035 articles were further evaluated, 455 of which were excluded for being systematic or retrospective reviews. The remaining 580 full-text articles were assessed, and after excluding studies where PD and/or AD were not the primary variable of interest, 52 studies were included in the summary tables. A PRISMA flow chart showing the study selection at each stage is detailed in Figure 1. Twenty-five studies explored the correlation between PD and AD through clinical research (Table 1). The studies were undertaken in the following regions: China *n* = 3, Finland *n* = 1, Germany *n* = 1, India *n* = 1, Italy *n* = 1, Japan *n* = 1, Korea *n* = 3, Pomerania *n* = 1, Spain *n* = 1, Sweden *n* = 1, Turkey *n* = 1, United Kingdom *n* = 1, USA *n* = 6, Mendelian randomization studies = 3. The highest number of studies were carried out in USA (*n* = 6), Korea (*n* = 3) and China (*n* = 3). And the studies included 7 case-control studies, 8 cohort studies, 6 cross-sectional studies, 3 Mendelian randomization studies and 1 quasi-experimental study. Twenty-four studies explored the potential mechanism underlying the correlation between PD and AD (Table 2). And the studies included 6 clinical studies conducted in Austria, the United Kingdom, China, Japan, Spain and the United States, 14 animal experiments and 4 cell experiments. Among the 6 clinical studies, 3 were cross-sectional studies, 2 were case-control studies and 1 Cross-sectional study. Three animal studies reported treatment progress based on the potential mechanism underlying the correlation between PD and AD (Table 3).

## 4 Discussion

This review systematically summarizes the existing evidence on the association between PD and AD, highlighting potential biological mechanisms underlying this relationship and discussing the implications of periodontal management in AD prevention and treatment. While accumulating epidemiological and experimental studies suggest a link between PD and an increased risk of cognitive decline, the causal relationship remains unclear. Further well-designed longitudinal studies and mechanistic investigations are required to establish a definitive connection and explore targeted interventions.

### 4.1 The correlation between PD and AD

PD has been identified as a chronic inflammatory condition associated with systemic health consequences, including cardiovascular disease, diabetes, and more recently, neurodegenerative disorders such as AD (D'Aiuto et al., 2025). Epidemiological studies have reported that individuals with PD exhibit a higher prevalence of AD and mild cognitive impairment (MCI) (Schwahn et al., 2022; Chen et al., 2017; Holmer et al., 2018). Several cohort and case-control studies have found that markers of PD (Syrjälä et al., 2012; Ma et al., 2022; Schwahn et al., 2022; Choi et al., 2019; Carballo et al., 2023; Fu et al., 2023; Ide et al., 2016; Panzarella et al., 2022; Noble et al., 2014; Yoo et al., 2023; Laugisch et al., 2021; Saito et al., 2022; Karaduran et al., 2023), such as clinical attachment loss and probing depth, are significantly associated with poorer cognitive performance. However, due to the observational nature of most studies, the directionality and causality of this association remain to be fully established.

Some studies propose that PD may contribute to AD pathogenesis by exacerbating neuroinflammation and promoting amyloid-beta (Aβ) deposition (Karaduran et al., 2023). Conversely, it is also plausible that AD-related cognitive decline leads to compromised oral hygiene, thereby increasing susceptibility to PD (Syrjälä et al., 2012; Martande et al., 2014). The bidirectional nature of this relationship warrants further investigation, particularly through longitudinal cohort studies and Mendelian randomization analyses to determine whether PD plays a causal role in AD progression.

### 4.2 Potential mechanisms of PD and AD

#### 4.2.1 PD and Aβ/tau protein aggregation

According to the amyloid hypothesis, abnormal accumulation of Aβ in specific brain regions triggers microglia-mediated inflammation, leading to neuronal damage and ultimately AD. PD may exacerbate this process by inducing systemic inflammation and allowing microbial components to cross the blood–brain barrier (BBB), thereby promoting Aβ deposition (Ahmad et al., 2019). In addition, PD may induce abnormal tau phosphorylation and the formation of neurofibrillary tangles (NFTs), further accelerating AD pathology (Ryder and Xenoudi, 2021). Several studies have demonstrated that PD enhances microglial activation (Díaz-Zúñiga et al., 2019; Kantarci et al., 2020; Ilievski et al., 2018; Qian et al., 2021) induces neuroinflammatory responses, and leads to neuronal injury, while animal models of PD also exhibit tau hyperphosphorylation and NFT formation (Ilievski et al., 2018). Thus, interventions aimed at mitigating excessive microglial activation might offer potential benefits in slowing AD progression.

#### 4.2.2 The role of Porphyromonas gingivalis (Pg) in AD

*Pg* is a key periodontal pathogen implicated in PD and has been detected in the brain tissues of AD patients (Poole et al., 2013). Studies (Sato et al., 2022; Gu et al., 2020) have shown that *Pg* lipopolysaccharide (LPS) is present in AD brains, whereas it is absent in cognitively normal individuals. Animal experiments (Ilievski et al., 2018; Bahar et al., 2021) further indicate that oral infection with *Pg* can induce AD-like neuropathological changes, including Aβ deposition, neuroinflammation, and neuronal loss. However, there is still debate regarding the specific toxic components of *Pg*: while some studies attribute the neurotoxicity to *Pg* LPS (Sato et al., 2022; Gu et al., 2020), others suggest that phosphoglycerol dihydroceramide produced by *Pg* may play a more crucial role (Yamada et al., 2020). Further research is required to pinpoint the primary virulence factor of *Pg* in AD pathogenesis and to develop targeted therapeutic strategies against *Pg* infection.

#### 4.2.3 Microbial invasion routes

Multiple routes may allow periodontal pathogens to invade the central nervous system (CNS). Periodontal pathogens, particularly *Pg*, produce virulent factors: specifically, gingipains: that compromise the integrity of the blood-brain barrier, allowing bacteria and inflammatory mediators to enter the central nervous system (Kim and Pang, 2025). Oral pathogens may also migrate directly to the brain via cranial nerve routes, such as the trigeminal or olfactory nerves (Sakane et al., 2020). Additionally, secondary routes—particularly the gastrointestinal tract—may be involved. PD-induced oral dysbiosis can alter gut microbial composition, promoting systemic inflammation and neuroinflammation through the gut–brain axis (Xue et al., 2020; Lu et al., 2022). These pathways likely act in combination, enhancing CNS vulnerability in the context of chronic oral infection.

#### 4.2.4 Periodontal microbiome dysbiosis and metabolic abnormalities

Beyond direct microbial invasion, PD-associated dysbiosis alters host metabolism. Metabolomic studies have identified specific PD-related metabolites (e.g., galactinol, D-mannitol) that predict AD progression (Leblhuber et al., 2020; Qiu et al., 2024). Gut microbiome disturbances induced by PD have also been linked to neuroinflammation and cognitive decline in animal models (Xue et al., 2020; Lu et al., 2022). However, clinical validation remains limited, and further studies are needed to confirm whether microbiome-targeted therapies can mitigate AD risk.

### 4.3 The correlation between PD treatment management and AD

Given that there are currently no disease-modifying therapies for AD and that existing treatments offer only transient symptomatic relief, early intervention is critical (Di Santo et al., 2013). PD has been proposed as a modifiable risk factor for AD, suggesting that its treatment might provide a novel approach for AD prevention and management (Dominy et al., 2019). Several studies have demonstrated that periodontal therapy—such as scaling and root planning—may reduce systemic inflammatory markers and potentially improve cognitive outcomes.

For instance, Schwahn et al. (2022) indicated that periodontal treatment might mitigate AD-related brain atrophy, while Saito et al. (2022) reported a lower risk of AD among individuals who received long-term periodontal care. Additionally, preclinical studies (Zhao et al., 2023) have shown that gingipain inhibitors can reduce Aβ production, dampen neuroinflammation, and protect hippocampal neurons, and that probiotics such as Nisin can ameliorate AD-like pathology by restoring microbial balance. More recently, nanotechnology-based strategies have emerged; an animal study (Liu et al., 2025) demonstrated that *Pg*-stimulated macrophage membrane-coated platinum nanoclusters (*Pg*-M-PtNCs) exhibited good biocompatibility, effectively inhibited *Pg* growth, and reduced bacterial load and neuronal injury *in vivo*, thereby improving AD-like cognitive impairment. Collectively, these findings highlight the therapeutic potential of periodontal treatment and novel experimental approaches, although their efficacy requires confirmation in large-scale clinical trials.

### 4.4 Conflicting findings and limitations

Despite accumulating evidence, inconsistencies remain. Some studies report no significant association between PD and AD after adjusting for confounders such as age, education, and comorbidities, suggesting that residual confounding cannot be excluded. Additionally, the majority of studies are cross-sectional or retrospective, which limits causal interpretation. Heterogeneity in study populations, PD diagnostic criteria, and cognitive assessment tools further complicates comparisons across studies. It is important to note that this scoping review provides a valuable mapping of existing evidence but offers a lower level of evidence compared to systematic reviews or meta-analyses. This distinction should be kept in mind when interpreting the findings. Another limitation is language and publication bias: this review only included English-language and peer-reviewed articles, excluding gray literature, which may have led to omission of relevant data. Incorporating gray literature in future reviews may reduce bias and provide a more balanced synthesis.

### 4.5 Future directions

Future research should prioritize longitudinal and interventional studies to clarify causality and mechanisms. Standardization of PD and AD diagnostic criteria, along with harmonized outcome measures, would facilitate cross-study comparisons. Integration of microbiome profiling, metabolomics, and neuroimaging could provide new insights into biological pathways. Importantly, evaluating periodontal treatment as part of multidomain AD prevention strategies warrants exploration in clinical trials.

## 5 Conclusion

This review synthesizes current evidence on the association between PD and AD. Epidemiological studies consistently demonstrate that individuals with PD have a higher prevalence of cognitive decline and AD, although most available data are observational and causality has not been established. Experimental studies provide mechanistic insights, suggesting that PD may contribute to AD pathology through chronic systemic inflammation, amyloid-β and tau aggregation, neuroinvasion of *Pg*, and oral–gut–brain microbiome dysregulation. Limited but emerging evidence also indicates that periodontal treatment may attenuate systemic inflammation and potentially improve cognitive outcomes.

Taken together, these findings highlight PD as a potential modifiable risk factor for AD. While current evidence supports biological plausibility and a consistent association, robust longitudinal and interventional studies are still needed to determine whether improving periodontal health can reduce the incidence or progression of AD.

## Data Availability

The original contributions presented in the study are included in the article/Supplementary material, further inquiries can be directed to the corresponding author.
