# Supplementary material for: Association between periodontal disease and Alzheimer's disease: a scoping review
Source: Front Aging Neurosci. 2025 Oct 15;17:1588008. doi: 10.3389/fnagi.2025.1588008 (PMC12568533; doi:10.3389/fnagi.2025.1588008)
Supplement: Supplementary file 2 [file Supplementary_file_2.docx]

**Protocol: Association between Periodontal Disease and Alzheimer’s Disease — A Scoping Review**

**1. Title**

Association between periodontal disease and Alzheimer’s disease: a scoping review protocol

**2. Background and Rationale**

Alzheimer’s disease (AD) is a progressive neurodegenerative disorder characterized by cognitive decline, memory impairment, and behavioral disturbances, representing the most common cause of dementia worldwide. Neuropathological hallmarks include β-amyloid (Aβ) plaques, neurofibrillary tangles composed of hyperphosphorylated Tau protein, synaptic dysfunction, and chronic neuroinflammation mediated by activated glial cells and proinflammatory cytokines.

Periodontal disease (PD) is a chronic, multifactorial inflammatory condition initiated by oral microbial dysbiosis, leading to tissue destruction, alveolar bone loss, and systemic inflammation. Evidence suggests that periodontal pathogens and their virulence factors can disseminate via the bloodstream, cross the blood–brain barrier, and contribute to neuroinflammation and neuronal injury.

Emerging research has proposed the existence of a “PD–brain axis,” whereby chronic periodontal inflammation and microbial dissemination may contribute to AD pathogenesis. Despite the growing body of evidence, findings remain heterogeneous and fragmented across clinical, epidemiological, and mechanistic studies. A scoping review is therefore warranted to systematically map the extent, range, and nature of existing research on this topic, identify key mechanisms, and highlight gaps for future research.

**3. Objectives**

The aim of this scoping review is to:

1. Map the existing evidence on the association between periodontal disease and Alzheimer’s disease.

(2) Identify proposed biological and pathological mechanisms linking PD and AD.

(3) Summarize study designs, populations, and outcomes used in the literature.

(4) Highlight research gaps and implications for prevention and therapy.

**4. Research Question**

Primary question: What is the current state of evidence regarding the association between periodontal disease and Alzheimer’s disease?

Secondary question: What biological mechanisms have been proposed to explain the PD–AD connection?

**5. Methods**

**5.1 Study Design**

This study will be conducted as an exploratory scoping review, following the methodological framework of Arksey and O’Malley, further refined by the Joanna Briggs Institute (JBI), and reported in accordance with PRISMA-ScR guidelines.

**5.2 Eligibility Criteria**

Inclusion criteria:

1. Study types: Original research articles (clinical studies, epidemiological studies, experimental animal studies, in vitro studies if related to mechanisms).
2. Population: Human participants with PD and/or AD, or animal models of PD/AD.
3. Concept: Explicit assessment of the association between periodontal disease and (4)Alzheimer’s disease, or investigation of potential mechanistic pathways.

(5)Language: English.

(6)Time frame: January 2004 – February 2024.

**Exclusion criteria:**

1. Systematic reviews, meta-analyses, and narrative reviews.
2. Conference abstracts without full-text availability.
3. Case reports or studies with insufficient methodological detail.

**5.3 Information Sources**

(1) Electronic databases:PubMed, EMBASE, Cochrane Central Register of Controlled Trials

(2) Additional sources:Reference lists of included studies, Citation tracking of key articles.

(3) Gray literature will not be included in this review; this decision is based on the primary focus on peer-reviewed evidence to ensure methodological rigor and comparability. This is acknowledged as a limitation.

**5.4 Search Strategy**

A comprehensive search strategy combining Medical Subject Headings (MeSH) and free-text terms in title/abstract fields will be used. Boolean operators will be applied (“OR” within each concept group; “AND” between PD and AD concepts).

Example PubMed search string:(Periodontal Diseases[Mesh] OR Disease, Periodontal[Title/Abstract] OR Diseases, Periodontal[Title/Abstract]

OR Periodontal Disease[Title/Abstract] OR Parodontosis[Title/Abstract] OR Parodontoses[Title/Abstract] OR Pyorrhea Alveolaris[Title/Abstract])

AND (Alzheimer Disease[Mesh] OR Alzheimer Dementia[Title/Abstract] OR Alzheimer Dementias[Title/Abstract] OR Dementia, Alzheimer[Title/Abstract] OR Alzheimer's disease[Title/Abstract] OR Dementia, Senile[Title/Abstract] OR Senile Dementia[Title/Abstract] OR Dementia, Alzheimer Type[Title/Abstract] OR Alzheimer Type Dementia[Title/Abstract] OR Alzheimer-Type Dementia[Title/Abstract]). The strategy will be adapted for EMBASE and Cochrane Central.

**5.5 Study Selection**

(1) Records will be imported into EndNote for reference management and duplicate removal.

(2) Two independent reviewers will screen titles and abstracts against eligibility criteria.

(3) Full texts will be assessed for inclusion, with discrepancies resolved through discussion or a third reviewer.

**5.6 Data Extraction**

Data will be charted using a standardized extraction form including:

1. Bibliographic details (author, year, country).

(2) Study design and population (human/animal, sample size).

(3) Study objectives.

(4) Type of periodontal assessment and Alzheimer’s disease evaluation.

(5) Reported associations and mechanisms.

(6) Key outcomes and conclusions.

**5.7 Data Presentation**

Findings will be presented descriptively in tables and figures, summarizing study characteristics, methodological approaches, and proposed mechanisms. Thematic synthesis will be used to group findings into categories such as:Epidemiological evidence of PD–AD associations.Mechanistic evidence (e.g., systemic inflammation, blood–brain barrier disruption, microbial dissemination, neuroinflammation). Gaps and future research needs.

**6. Ethics and Dissemination**

This review does not involve primary data collection and thus does not require ethical approval. Results will be disseminated through peer-reviewed publication and conference presentations.

**7. Timeline**

Literature search: February 2024.

Study screening and selection: March–April 2024.

Data extraction and analysis: May–June 2024.

Manuscript preparation: July–August 2024.
